# Highly Conductive Al/Al Interfaces in Ultrafine Grained Al Compact Prepared by Low Oxygen Powder Metallurgy Technique

**DOI:** 10.3390/nano11051182

**Published:** 2021-04-30

**Authors:** Dasom Kim, Yusuke Hirayama, Zheng Liu, Hansang Kwon, Makoto Kobashi, Kenta Takagi

**Affiliations:** 1 Department of Materials Process Engineering, Nagoya University, 1 Furocho, Chikusa, Nagoya 464-8603, Japan; dasom.kim@f.mbox.nagoya-u.ac.jp (D.K.); kobashi.makoto@material.nagoya-u.ac.jp (M.K.); 2 Magnetic Powder Metallurgy Research Center, National Institute of Advanced Industrial Science and Technology, 2266-98 Anagahora, Shimoshidami, Moriyama, Nagoya 463-8560, Japan; k-takagi@aist.go.jp; 3 Innovative Functional Materials Research Institute, National Institute of Advanced Industrial Science and Technology, 2266-98 Anagahora, Shimoshidami, Moriyama, Nagoya 463-8560, Japan; liu-z@aist.go.jp; 4 Department of Materials System Engineering, Pukyong National Univerisity, 365, Sinseon-ro, Nam-gu, Busan 48547, Korea; kwon13@pknu.ac.kr

**Keywords:** aluminum, nanopowder, low oxygen powder metallurgy, electrical conductivity, mechanical property, ultrafine grain, conductive interface

## Abstract

The low oxygen powder metallurgy technique makes it possible to prepare full-dense ultrafine-grained (UFG) Al compacts with an average grain size of 160 nm by local surface bonding at a substantially lower temperature of 423 K from an Al nanopowder prepared by a low oxygen induction thermal plasma process. By atomic level analysis using transmission electron microscopy, it was found that there was almost no oxide layer at the Al/Al interfaces (grain boundaries) in UFG Al compact. The electrical conductivity of the UFG Al compact reached 3.5 × 10^7^ S/m, which is the same level as that of the cast Al bulk. The Vickers hardness of the UFG Al compact of 1078 MPa, which is 8 times that of the cast Al bulk, could be explained by the Hall–Petch law. In addition, fracture behavior was analyzed by conducting a small punch test. The as-sintered UFG Al compact initially fractured before reaching its ultimate strength due to its large number of grain boundaries with a high misorientation angle. Ultimate strength and elongation were enhanced to 175 MPa and 24%, respectively, by reduction of grain boundaries after annealing, indicating that high compatibility of strength and elongation can be realized by appropriate microstructure control.

## 1. Introduction

Bonding is an indispensable technology for establishing and functioning structures ranging from delicate components such as precision parts for home appliances to parts such as used in civil engineering, architecture, and shipbuilding. Conventional bonding basically involves high-temperature processes. In particular, the processes used in metal bonding, such as the solid-state bonding techniques of diffusion bonding [1,2], fusion welding [3], friction welding [4], and brazing [5], require holding at a high temperature for an extended time to achieve an interface without voids. Low-temperature bonding has several merits, including an increased degree of freedom in selecting the bonding target materials, reduced energy consumption, lower thermal strain, and suppressed interfacial reactants, resulting in improved mechanical strength of the bonded body.

When considering room temperature bonding methods, surface-activated bonding (SAB) is one interesting approach [6,7]. In SAB, the surface contamination that is always present on metal surfaces is removed in a high vacuum. A drastic reduction in the required bonding temperature is realized by applying a certain pressure between the activated metal surfaces. Bonding of similar metals [6,8] and dissimilar metals [9,10] by SAB has already been reported. SAB-bonded bodies are mainly used in manufacturing semiconductor devices. In this type of bonding, an atomically flat surface is an essential requirement, and surface cleaning is performed by a milling technique such as Ar milling or chemical mechanical polishing. As one drawback, the range of applications is limited, as a high-vacuum device is required to prevent redeposition after surface cleaning. Nevertheless, the results achieved with SAB provide helpful guidance for future development. They demonstrate that a high bonding temperature is unnecessary, and atomic-level bonding is possible under near-room temperature conditions if the surface is activated and atomically flat.

One technique for handling a large amount of bonding is powder metallurgy, which produces bulk bodies (compacts) from metal powder. With the present powder metallurgy technology, bonding (sintering) is performed locally at the surface of each particle at a temperature over 70% of the melting temperature [11,12]. The bonding interface strongly influences the properties of the bulk. Methods for lowering the sintering temperature include increasing surface energy by reducing the particle size [13] and direct bonding using bare particles with a contaminant-free surface [14,15]. When bonding metals can be reduced by hydrogen, a clean surface comparable to that by SAB can be obtained by annealing in a hydrogen atmosphere, which enables sintering at a lower temperature [16]. However, if the metal cannot be reduced by hydrogen, it is difficult to remove the contamination film existing on the surface of each metal particle. Aluminum (Al) is known to be one of the most oxidizable metals, which means a strong contamination film which cannot be removed by hydrogen is formed on the surface. Therefore, it has been reported that a sintering temperature of 823 K or higher is required in order to prepare a dense Al bulk from Al nanopowder, as this temperature is essential to destroy the surface oxide film [17,18].

If it is possible to develop a powder metallurgy process that enables low temperature bonding comparable to that in SAB, not only for Al but also for other metals, the range of applications can be expanded to higher resolution metal 3D printing [19,20] and fabrication of microdevices by nanoimprinting [21,22]. In application to metal matrix composites, use of metal nanopowders can significantly increase the degree of dispersion when mixed with other nano-sized reinforcing agents such as CNT and graphene [23,24], and the reaction between the reinforcing agent and the metal matrix can be controlled in detail by controlling the temperature during densification, resulting in higher properties. This suggests the possibility of developing new materials and/or devices by developing metal nanopowders that are lower temperature bonding.

Recently, we developed an innovative Al nanopowder called “bare” Al nanopowder by the low oxygen-induction thermal plasma (LO-ITP) process, which could realize ultrafine-grained (UFG) Al bulk with highly conductive Al/Al interfaces at even room temperature of 0.3 $T_{m}$ [25], indicating that the oxide layer at a grain boundary was negligibly thin for the electron conduction. This result also suggested the possibility of preparing new functional materials with enhanced properties, as described in the previous paragraph, by a low oxygen powder metallurgy technique. In order to demonstrate this possibility, in this paper, we prepared UFG Al bulks from the bare Al nanopowder under a controlled atmosphere and systematically investigated the effects of pressure and temperature on densification. The Al/Al interfaces in bulk were analyzed locally by atomic-level observation using high-resolution STEM, since the interface influences properties. As an average performance, the electrical and mechanical properties were evaluated. We proposed a microstructure-controlled Al bulk that simultaneously achieves high electrical conductivity, strength, and elongation based on the results.

## 2. Materials and Methods

### 2.1. Preparation of Al Nanopowder

Al nanopowder was prepared by the LO-ITP process [25] developed based on the fundamental TP40020NPS ITP system (JEOL Co., Ltd., Tokyo, Japan) and a TP-99010FDR powder feeder (JEOL Co., Ltd., Tokyo, Japan). The oxygen level was controlled to below 0.5 ppm. The Al raw powder (20 µm, purity 99.99%, oxygen level 0.095 wt%, Kojundo Chemical Lab. Co., Ltd., Saitama, Japan) was introduced through the powder feeder after introducing Ar gas with a process pressure of 70 KPa. The powder feed and Ar carrier gas rates were 0.3 g/min and 3 L/min, respectively. The prepared Al nanopowder was collected and stored in a vial filled with heptane, and then dispersed using an ultrasonic homogenizer (UH-150, SMT Corp, Sandy Hook, CT, USA) for a total of 100 min with actual working time efficiency of 30% in a glovebox to prevent oxidation. The Al nanopowder was observed by field emission scanning electron microscopy (FE-SEM, JSM-7800F, JEOL Co., Ltd.) without exposure to the air. The particle size distribution was determined from the measured diameter of observed 500 Al particles in the FE-SEM images. The average particle size was estimated by measuring the surface area of the Al nanopowder by the Brunauer–Emmett–Teller measurement (BET, 3 Flex Physisorption, Micromeritics Instrument Corp. Norcross, GA, USA) with nitrogen gas. As a reference powder for comparison with the LO-ITP processed Al nanopowder without air exposure (denoted as “unexposed Al nanopowder”), an unexposed Al nanopowder was exposed to 1% O_2_-Ar gas at room temperature (RT) for 12 h (denoted as “exposed Al nanopowder”). The oxygen weight fraction of the unexposed and exposed Al nanopowders was measured with an oxygen analyzer (EMGA-620W, Horiba Ltd. Kyoto, Japan). To analyze the oxygen content of the unexposed Al nanopowder without further oxidization during the analysis, the Al nanopowder was enclosed in a Ni capsule in the glovebox, and then carried to the analyzer in a transfer vessel filled with heptane. The heptane was evaporated before measurement in the analyzer.

### 2.2. Consolidation of Al Nanopowder

The prepared Al nanopowder was loaded into a tungsten carbide mold with a diameter of ø 6 or 8 mm in a glovebox, and the mold was mounted in the chamber of a current sintering machine (LABOXTM-625F-GH, Sinter Land Inc. Nagaoka, Japan) combined with a glovebox to avoid exposure to air. The mold containing the unexposed or exposed Al nanopowders was compacted at room temperature (RT) or sintered at 423 K or 673 K under various uniaxial pressures of 100, 300, 600, 900 and 1200 MPa in a vacuum. When conducting sintering, the heating rate was 40 K/min, and holding time was 1 min at the sintering temperature.

### 2.3. Characterization

The actual density of the prepared Al compacts was measured by the Archimedes method. The correct relative density was calculated by the rule of mixture with the volume fraction of Al and Al oxides, which was calculated using the measured oxygen weight. Assuming all oxygen is present as Al_2_O_3_, the volume fraction of Al_2_O_3_ ($V_{Al_{2}O_{3}}$) was calculated by Equation (1).

(1) $$V_{Al_{2}O_{3}}=W_{O}\cdot M_{Al_{2}O_{3}}/\left\{W_{O}\cdot M_{Al_{2}O_{3}}+\rho_{Al_{2}O_{3}}\cdot \left(3M_{O}\cdot W_{Al}-2M_{Al}\cdot W_{O}\right)/\rho_{Al}\right\}$$

where $W_{O}$ and $W_{Al}$ are the weight percent of O and Al ($W_{O}+W_{Al}=$100 wt%). $M_{Al_{2}O_{3}}$, $M_{O}$ and $M_{Al}$ are the molar mass of Al_2_O_3_ (102 g/mol), O (16 g/mol) and Al (27 g/mol), respectively. $\rho_{Al_{2}O_{3}}$ and $\rho_{Al}$ are the density of Al_2_O_3_ (3.96 g/cm^3^) and Al (2.70 g/cm^3^).

With the $V_{Al_{2}O_{3}}$ and expected mean particle size (D), the average thickness of oxide film ($t_{Al_{2}O_{3}})$ of unexposed and exposed Al nanopowders was calculated by Equation (2).

(2) $$t_{{Al}_{2}O_{3}}=\{D(1-\sqrt[{3}]{1-V_{{Al}_{2}O_{3}}})\}/2$$

To prepare thin samples for the microstructure analysis, a Ga-focused ion beam was used. The microstructure was analyzed using a JEM-ARM200F ACCELARM (cold FEG, JEOL Ltd. Tokyo, Japan) equipped with double CEOS Cs correctors (ASCOR system for scanning transmission electron microscopy (STEM)). For elemental mapping, an energy-dispersive X-ray spectroscope (EDS, JED-2300, JEOL Ltd. Tokyo, Japan) was used. The transmission Kikuchi diffraction (TKD) map and misorientation map superimposed on the band contrast (BC) map was obtained by Nordlys Nano (Oxford instruments).

Vickers hardness and small punch tests were conducted to characterize the mechanical properties of the Al compacts. Vickers hardness was measured using a hardness tester (HV-114, Mitutoyo Corp. Kawasaki, Japan) with a load of 1 kg for 15 s. The small punch (SP) test was conducted using a universal testing machine (AG-50kN, Shimadzu Corp, Kyoto, Japan). In the schematic illustration of the SP test apparatus [26], the punch radius was 1.19 mm, and the diameter of the receiving die hole was 4 mm. The displacement rate was set at 0.1 mm/min. A cylindrical specimen (diameter: 8 mm, thickness: 0.350 ± 0.002 mm) was used in the SP test. The electrical conductivity of the Al compact was measured by the four-probe method using a Loresta-GX MCP-T700 (Mitsubishi Chemical Analytech Co., Ltd, Tokyo, Japan) at room temperature.

## 3. Results and Discussions

**Figure 1:**
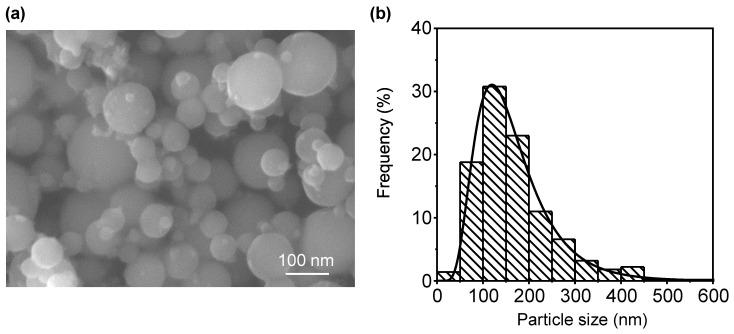
(**a**) FE-SEM image of Al nanopowder prepared by LO-ITP process and (**b**) histogram of Al particle size distribution.

In this study, the LO-ITP process was used to prepare bare Al nanopowder. The Al particles were spherical, as shown in Figure 1a. According to the histogram of the measured Al particle size distribution shown in Figure 1b, the mean particle size was 165 nm with a standard deviation (σ) of 81 nm. In order to evaluate the overall particle size, a BET measurement was conducted. As the measured specific surface area was 14.7 m^2^/g, the average particle size was estimated to be 152 nm with the theoretical density of Al (2.7 g/cm^3^). The particle size obtained from the BET measurement substantially coincided with that in the FE-SEM images, implying that the prepared Al nanopowder was composed of Al nanoparticles isolated without necking during the LO-ITP process.

**Figure 2:**
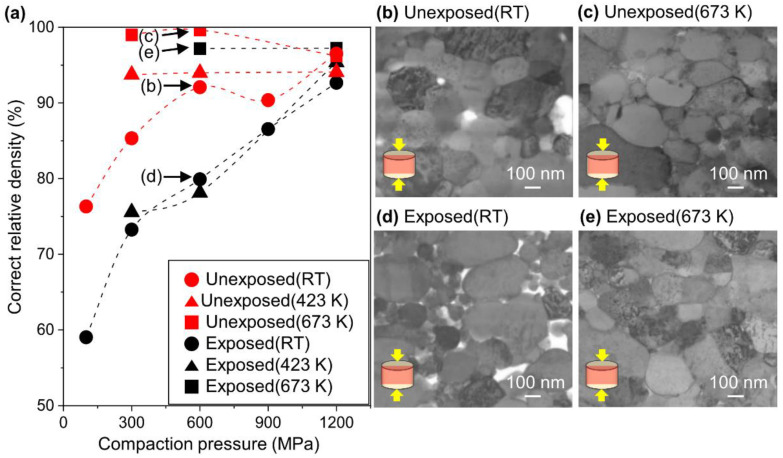
(**a**) Correct relative density of Al compacts as a function of compaction pressure. The dotted lines are a guide for the eye. (**b**–**e**) Annular bright-field (ABF)-STEM images of unexposed and exposed Al compacts pressed under 600 MPa along a direction of the arrows (insets) with and without sintering at 673 K. (**b**) Unexposed Al compact pressed at RT, (**c**) unexposed Al compact sintered at 673 K, (**d**) exposed Al compact pressed at RT, and (**e**) exposed Al compact sintered at 673 K.

To understand the densification behavior of the obtained Al nanopowder, the compaction pressure and sintering temperature were varied. Figure 2a shows the correct relative density of the Al compacts consolidated from the unexposed and exposed Al nanopowders against the compaction pressure at room temperature, 423 K and 673 K. To investigate the microstructural difference between the unexposed and exposed Al compacts, the cross-sections of the Al compacts prepared under the pressure of 600 MPa were observed by ABF-STEM, as shown in Figure 2b–e. The direction of uniaxial compaction pressure was parallel to the longitudinal direction of the images.

First, the effect of compaction pressure at room temperature will be discussed. For both the unexposed and exposed Al compacts, the correct relative density increased as the compaction pressure increased. Still, the density of the unexposed Al compacts was higher than that of the exposed Al compacts under the same compaction pressure. It was found that the unexposed Al nanopowder undergoes plastic deformation and shape fitting even at room temperature (Figure 2b). In contrast, the exposed Al nanopowder maintained its spherical shape without shape fitting, resulting in many voids between the particles (Figure 2d).

Next, the effect of the temperature will be discussed. The correct relative density of the unexposed Al compact was already saturated at 423 K, even under the compaction pressure of 300 MPa. In contrast, the exposed Al compact was not fully densified below 423 K, and finally reached saturation at 673 K under the pressure of 600 MPa.

The degree of plastic deformation was quantified by calculation from the ratio of the particle diameter in the uniaxial direction ($D_{y}$) and in the orthogonal direction to the compaction pressure direction ($D_{x}$) using images as shown in Figure 2b,d. As can be seen from Figure 1a, the average aspect ratio ($D_{x}/D_{y}$) of the as-TP Al nanopowder was 1.00, after cold compaction under 600 MPa, the average aspect ratios of the unexposed compact obtained from Figure 2b was 1.16, while that of the exposed Al compact was 1.09 (Figure 2d). This difference might be attributed to the oxide film on the Al particles. Since the flexural strength of Al_2_O_3_ is around 400 MPa and the load resistance of the oxide increases proportionally to the square of thickness, the unexposed Al nanopowder without an oxide film was deformed more easily under the same pressure. After sintering at 673 K, the aspect ratios of the unexposed and exposed Al compacts were approximately the same, being 1.18 for the unexposed Al compact (Figure 2c) and 1.17 for the exposed Al compact (Figure 2e), indicating that both the unexposed Al and exposed Al compacts have the same degree of plastic deformation.

**Figure 3:**
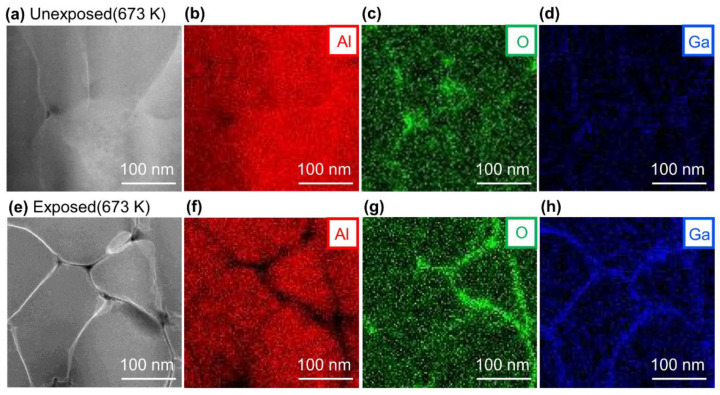
(**a**) high-angle annual dark-field (HAADF)-STEM image and EDS mapping results of (**b**) Al, (**c**) O, and (**d**) Ga of unexposed Al compact sintered at 673 K under 600 MPa for 1 min, and (**e**) HAADF-STEM image and EDS mapping result of (**f**) Al, (**g**) O and (**h**) Ga of exposed Al compact sintered at 673 K under 600 MPa for 1 min.

Figure 3a–d shows the HAADF-STEM images and EDS mapping images of the cross-section of the unexposed Al compact, and Figure 3e–h shows the results for the exposed Al compact sintered at 673 K under 600 MPa for 1 min. In the unexposed Al compact, the oxygen was mainly located at a triple junction (interface between three grains), and not at a grain boundary (interface between two grains) (Figure 3c), while oxygen was also detected at the grain boundaries in the exposed Al compact (Figure 3g). In the STEM-HAADF image, the white grain boundaries of the Al grains appear to be Ga accumulated during FIB processing since Ga has a larger Z value than Al. This can be confirmed from the Ga mapping images in Figure 3d,h. In sample preparation using FIB with a Ga source, it has been reported that Ga is accumulated at the grain boundaries [27]. Since Ga accumulates in the interface, it is easy to identify the locations of the grain boundaries.

**Figure 4:**
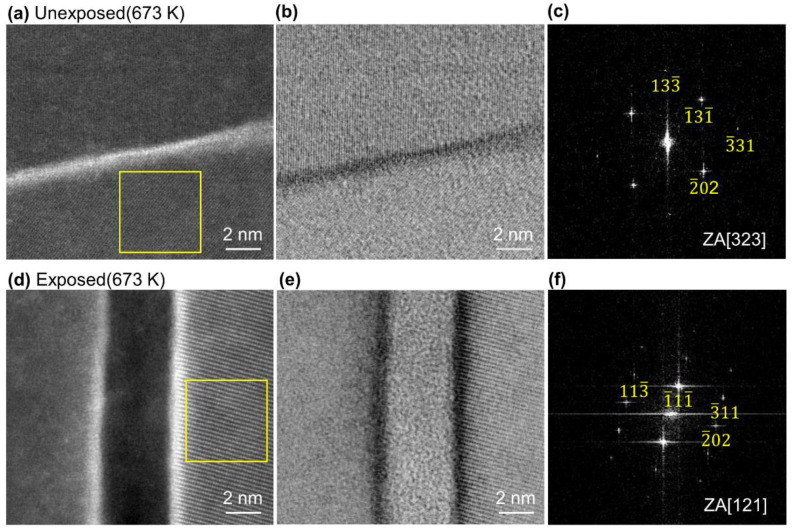
(**a**) HAADF-STEM image and (**b**) ABF-STEM image of unexposed Al compact sintered at 673 K under 600 MPa for 1 min, (**c**) fast Fourier transform (FFT) of the region indicated by yellow square in (**a**), (**d**) HAADF-STEM image and (**e**) ABF-STEM image of exposed Al compact sintered at 673 K under 600 MPa for 1 min showing the interface area between two Al grains, (**f**) FFT of the region indicated by yellow square in (**d**).

The results of further detailed observations of one arbitrary grain boundary of the Al grains are shown in Figure 4. The HAADF-STEM image of the unexposed Al compact in Figure 4a shows that this region is a grain boundary phase with the white contrast part (the region where Ga was accumulated). Lattice fringes can be more clearly seen from the ABF-STEM image (Figure 4b). Figure 4c shows a FFT image having a zone axis (ZA) of [323], obtained from the area surrounded by the yellow square in Figure 4a. From Figure 4a,b, it was found that there was almost no oxide layer at the grain boundary between the Al grains.

On the other hand, in the exposed Al compact, an oxide layer with a thickness of 4.2 nm between the Al grains, which show FFT image having a ZA of [121] (Figure 4f), was observed from the HAADF- and ABF-STEM images in Figure 4d,e. This oxide interface thickness in exposed Al compact was very consistent with that of the oxide phase obtained from previous reports [28]. Thus, we successfully demonstrated, for the first time, that almost no interface oxidation grain boundary; the clean interface of Al nanopowder was realized by handling in an extremely low oxygen environment, even when using the powder metallurgy technique. Especially when using Al nanopowder, which has a much higher specific surface area, this low oxygen process can be used to prepare Al compacts with clean, oxide-free interfaces like those obtained by SAB technique. As mentioned above, the SAB technique is quite difficult and inefficient to apply to powders with many surfaces.

Up to this point, we have discussed the local interfaces. Now we will discuss the average thickness of the oxide layer at the interfaces using the oxygen measurements of the Al nanopowder. Assuming that the contamination layer consists of an oxide film by the measured oxygen content on the particle surface, the oxide film thickness can be calculated by using the Equations (1) and (2). The oxygen content was determined to be 0.38 wt% for the unexposed Al nanopowder and 3.73 wt% for the exposed Al nanopowder. When these values are converted to the thickness of the oxide film on the Al particle surface, it can be estimated to be 0.15 ± 0.08 nm and 1.56 ± 0.84 nm, respectively. The small deviation of the film thickness obtained from the oxygen analysis and TEM observation is due to the fact that all Oxygen forms the crystalline phase of Al_2_O_3_, and the amount of oxides deposited at the triple junction was not considered. In any case, this result clearly demonstrates that the surface oxide film on Al particles can be drastically reduced by handling the Al nanopowder in a low oxygen atmosphere.

**Figure 5:**
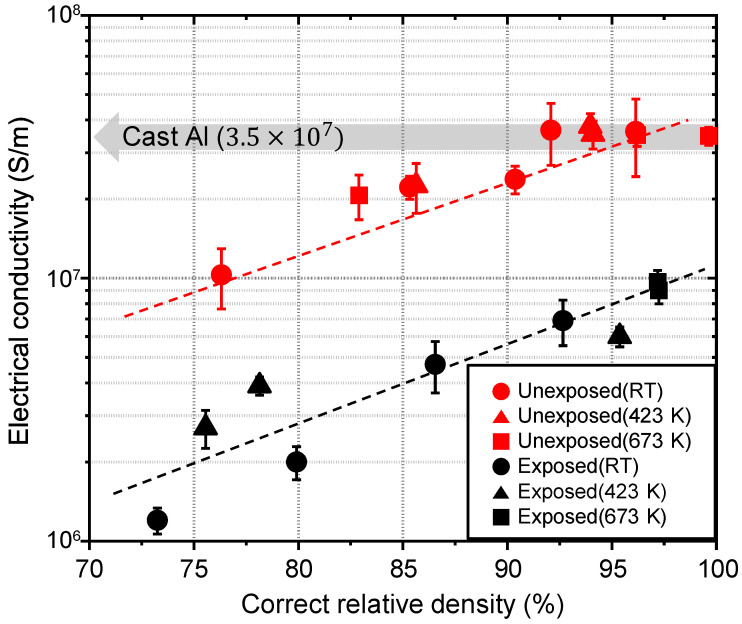
Electrical conductivity of cold-compacted or sintered unexposed and exposed Al compacts as a function of correct relative density.

Electric conduction as an average information was strongly influenced by the grain boundary state. The electrical conductivity of the unexposed and exposed Al compacts measured at room temperature was plotted as a function of the correct relative density, as shown in Figure 5. The electrical conductivity of both the unexposed and exposed Al compacts increased as the correct relative density increased. The absolute value of electrical conductivity of the exposed Al compacts was 4 to 8 times lower than that of the unexposed compacts at the same correct relative density. Finally, at full density, the electrical conductivity of the exposed and unexposed Al compacts reached 1.0 × 10^7^ S/m and 3.5 × 10^7^ S/m, respectively. The electrical conductivity of unexposed Al compact is comparable to that of the cast Al bulk.

Comparing the unexposed and exposed Al compacts, the difference in electrical conductivity is thought to result mainly from the thickness of the oxide layer at the grain boundaries. Basically, electrons are scattered at grain boundaries or impurities with high resistivity, and the electron mean free path is reduced. The electrical conductivity of Al compacts deteriorates greatly as the particle size decreases due to the high specific area of grain boundaries with oxide layers. However, when the oxide thickness is very thin, electrons can be transmitted through the barrier of the oxide layer by the so called “tunneling effect” [29]. The percentage of electron transmission through the barrier increases exponentially as the barrier thickness decreases. According to the Wentzel-Kramers-Brillouin (WKB) approximation equation using the barrier height of 3.0 eV for the Al oxide between Al particles [30,31], the transmission coefficient increases from 0% at the barrier thickness of 0.5 nm to 100% at the barrier thickness of 0.1 nm. This indicates that grain boundaries with a very thin oxide layer under 0.1 nm in thickness can function as gates for electrons, and deterioration of electrical conductivity can be prevented by preparing Al compact with grain boundaries with an oxide layer thickness of under 0.1 nm. Since the UFG Al compact prepared by using the low oxygen powder metallurgy process in this study achieved either no oxide layer or an extremely thin layer at the grain boundaries, the same level of electrical conductivity as in cast Al was obtained, even though the Al compact was composed of fine crystal grains with many grain boundaries that normally cause electrical resistance.

**Figure 6:**
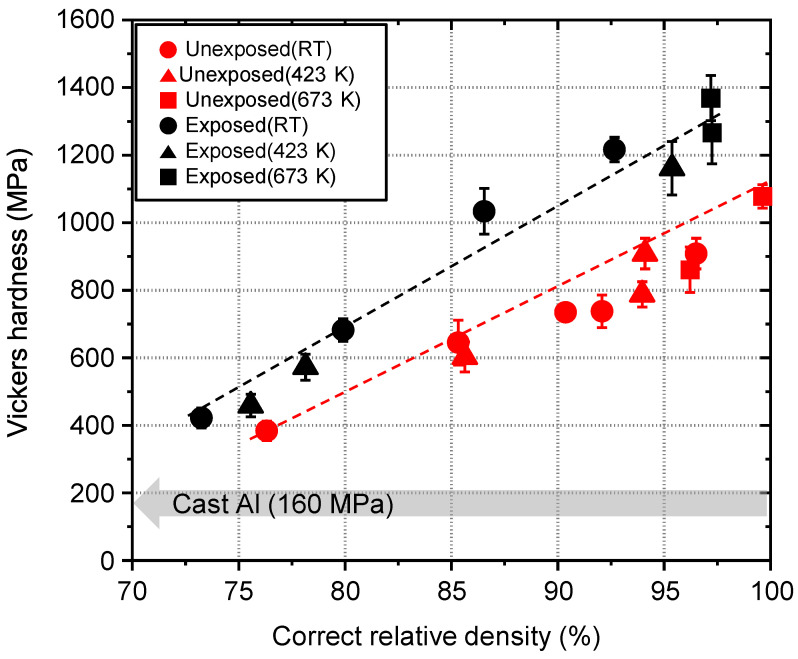
Vickers hardness of cold-compacted or sintered unexposed and exposed Al compacts as a function of correct relative density.

The grain boundary state influences the mechanical strength of the bulk body. The strength to plastic deformation of unexposed and exposed Al compacts were evaluated by the Vickers hardness test. The Vickers hardness of the unexposed and exposed Al compacts increased with increasing correct relative density and reached 1078 MPa and 1372 MPa at the correct relative density of 100% and 97%, respectively, as shown in Figure 6. The Vickers hardness values of the unexposed and exposed Al compacts were 7 and 9 times higher than that of the commercial cast Al (160 MPa), respectively. The difference between unexposed and exposed Al compacts are attributable to the presences of Al oxide at grain boundaries; (i) the Vickers hardness of the Al oxide is 100 times higher than that of Al, (ii) the flow stress could much increase due to thick oxide layer of exposed Al compact (the flow stress is proportional to grain boundary thickness squared [32]).

According to the Hall–Petch plot between hardness and grain size of UFG Al bulk prepared by severe plastic deformation (SPD) suggested by Matsui et al. [33] and Hayes et al. [34], the hardness of Al compact with the grain size of 160 nm estimated from the SEM image as shown in Figure 2 can be calculated to be 550 MPa, while the hardness of the unexposed UFG Al obtained in this experiment is 1078 MPa. Here, the grain size was estimated as an average value of the length in the uniaxial compression direction and the orthogonal to the compression direction. To understand this discrepancy, the misorientation angle (*θ*) of the grains, which is strongly dependent on mechanical properties, should be considered. Since the dislocation density increased with the increase of *θ* and is saturated at *θ* ~15°, high angle grain boundary (HAGB) has a higher grain boundary energy state than low angle grain boundary (LAGB).

**Figure 7:**
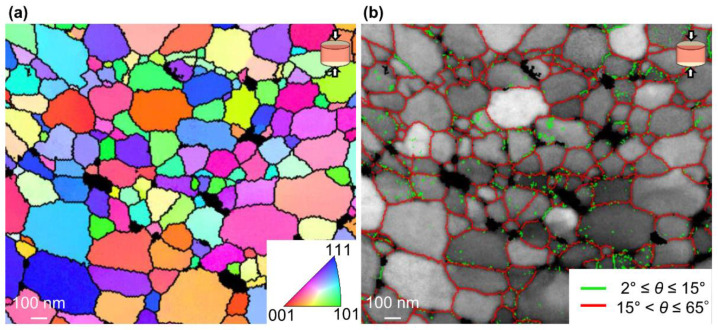
(**a**) TKD inverse pole figure (IPF) map and (**b**) misorientation map superimposed on BC map of unexposed Al compact sintered at 673 K under 600 MPa for 1 min.

Figure 7 shows a TKD IPF and misorientation map of the unexposed Al compact sintered at 400 °C under 600 MPa. According to the TKD IPF map in Figure 7a, the unexposed Al compact was composed of single-crystal grains with significantly varied crystal orientations. In Figure 7b, LAGB with misorientation *θ* ≤ 15° and HAGB with *θ* > 15° are shown by green and red lines, respectively, on the BC map. As expected, the grain boundaries were mainly composed of HAGBs, which indicates that almost all grain boundaries have a high energy state. On the other hand, the UFG Al bulks prepared by SPD typically have a significant amount of LAGBs [35,36,37]. Therefore, the unexposed Al compact has a higher Vickers hardness at the same grain size than UFG Al bulk prepared by SPD.

Since Vickers hardness is related to strength to plastic deformation, yield strength can be estimated, but the fracture behavior could not be discussed. In addition, materials with sub-micron size grains generally have higher strength but lower elongation [38]. Therefore, the small punch (SP) test was performed, as it is possible to evaluate strength and elongation even with small specimens by this technique. An Al specimen sintered at 673 K under 600 MPa for 1 min (denoted as “as-sintered Al”) and an Al specimen sintered at 673 K under 600 MPa for 1 min and then annealed at the 673 K for 30 min (denoted as “annealed Al”) were tested. As a reference to the SP test, pure Al film with a purity of 99.99% (AL-013522, The Nilaco Corp. Tokyo, Japan) was also used (denoted as “cast Al”).

**Figure 8:**
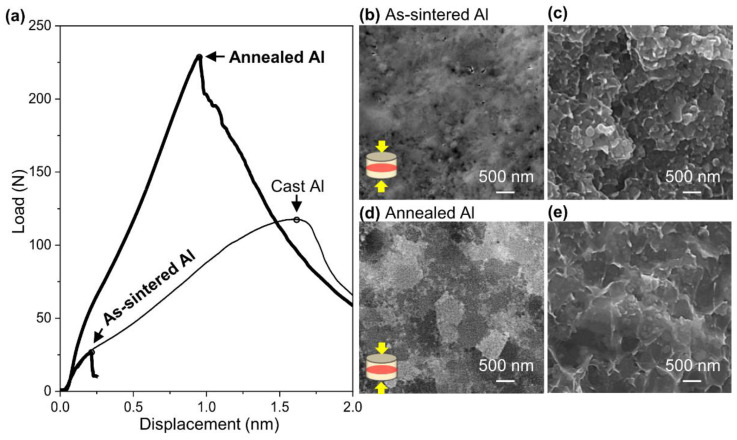
(**a**) Load-displacement curve of as-sintered, annealed Al compact and cast Al bulk used as reference (the small circles put on a curve at maximum load). FE-SEM images of (**b**) cross-section and (**c**) fracture surface after SP test of as-sintered Al, and (**d**) cross-section and (**e**) fracture surface after SP test of annealed Al.

As shown in Figure 8a, the cast Al used as a reference shows the typical curve of a ductile material [39]. Since the maximum load and displacement are supposed to be proportional to ultimate strength and elongation, respectively [26,39], the load of 118 N and displacement of 1.6 mm for the cast Al bulk were estimated to correspond to an ultimate strength of 90 MPa and elongation of 40% [40], respectively. The curve of the as-sintered Al failed at the load of 26 N and displacement of 0.2 mm, indicating ultimate strength of 20 MPa and elongation of 5%. Since the ultimate strength is much lower than the calculated yield strength ($\sigma_{y}$) of 359 MPa from Vickers hardness ($H_{V}$) according to estimation equation ($H_{V}\approx 3\cdot \sigma_{y}$) [41], the as-sintered Al may have fractured during elastic deformation. After annealing at 673 K for 30 min, the maximum load and displacement increased simultaneously to 230 N and 0.9 mm, respectively. Therefore, the ultimate strength and elongation of the annealed Al were estimated to be 175 MPa and 24%.

According to the microstructure observation of the as-sintered Al compact (Figure 8b) and annealed Al compact (Figure 8d), grain growth from 165 nm to 551 nm was observed, indicating that the number of grain boundaries decreased. In the observation of the fracture surface of the as-sintered Al compact (Figure 8c) and the annealed Al compact (Figure 8e), dimples with the same diameter as the grains were observed, as shown in Figure 8b,d, implying intergranular fracture. Since a crack propagates along grain boundaries in the intergranular fracture mode, the grain growth caused by annealing can contribute to preventing crack propagation and delaying fracture, and as a result, both strength and elongation increase. Therefore, the high compatibility of strength and elongation can be achieved easily by appropriate microstructural modification by a post-process such as annealing. Other mechanical properties such as compressive strength and shear strength are not discussed in this paper and will be reported in future work.

In this work, we proposed a new low oxygen powder metallurgy technique for preparing UFG Al compacts having enhanced strength and improved elongation, as a potential alternative to conventional SPD techniques which can realize an Al bulk with a UFG microstructure, such as Equal Channel Angular Pressing (ECAP) [42], High Pressure Torsion (HPT) [43], Cyclic Extrusion and Compression (CEC) [44] and Accumulative Roll Bonding (ARB) [45]. Since a UFG bulk can be produced by sintering in this process, the proposed technique is advantageous for handling shapes and sizes that have been difficult to produce by the conventional SPD techniques. Moreover, the obtained UFG bulk is relatively isotropic, since spherical particles are used as the starting material. This is one interesting feature that cannot be achieved by the other plastic deformation processes. Moreover, since Al nanopowder has an extremely thin oxide film, higher properties can be expected by improving dispersibility and advanced interface control by using the nanopowder as a precursor of a metal matrix composite material. Because the LO-ITP process can also be applied to the preparation of a wide range of nanopowders of binary and/or multiple alloy systems [46,47,48], it is also possible to expand this process, for example, to other Al [49] and Ti [50] systems used as structural materials.

## 4. Conclusions

UFG Al compacts were prepared from an unexposed Al nanopowder with less oxide film and prepared by the LO-ITP process. The unexposed Al nanopowder was fully densified at a substantially lower temperature of 423 K under a compaction pressure of 300 MPa. During consolidation, UFG Al compacts were prepared without grain growth by local surface sintering of the Al nanopowder. According to atomic-level observation by HR-STEM, it was found that there is almost no oxide layer at grain boundaries for the unexposed Al compact. Since the Al/Al grain boundaries are thin enough to work as a gate for electrons, the electrical conductivity of the unexposed Al compacts with the highly conductive grain boundary, which reached the same level as in the cast Al bulk. The hardness of the UFG Al compacts was up to 8 times higher than that of the commercial cast Al used as a reference due to the effect of particle refinement, which is explained by the Hall-Petch law. Although the strength measured by the SP test was lower than the expected strength due to the large area fraction of HAGBs, the strength could be enhanced to 175 MPa, while also increasing elongation to 24%, by controlling the grain boundary fraction through annealing. Thus, this study demonstrated the possibilities of the low oxygen powder metallurgy process, even when using nanoparticles with a large specific surface area, suggesting that the characteristics derived from the fine structure of the UFG bulk can be controlled.

## Data Availability

The data of this study are available within this article; further inquiries may be directed to the authors.
